# Mitochondrial DNA and local ecological knowledge reveal two lineages of leatherback turtle on the beaches of Oaxaca, Mexico

**DOI:** 10.1038/s41598-023-33931-4

**Published:** 2023-05-31

**Authors:** Carlos Abraham Castillo-Morales, Andrea Sáenz-Arroyo, Gabriela Castellanos-Morales, Lorena Ruíz-Montoya

**Affiliations:** 1 Departamento de Conservación de la Biodiversidad, El Colegio de la Frontera Sur (ECOSUR), Carretera Panamericana y Periférico Sur S/N, Barrio de María Auxiliadora, San Cristóbal de las Casas, Chiapas Mexico; 2 Centro de Ciencias de la Complejidad, Universidad Nacional Autónoma de México (C3-UNAM), Mexico City, Mexico; 3 Departamento de Conservación de la Biodiversidad, El Colegio de la Frontera Sur, Unidad Villahermosa (ECOSUR-Villahermosa), Carretera Villahermosa-Reforma km 15.5, Ranchería Guineo 2a sección, Villahermosa, Tabasco Mexico

**Keywords:** Ecology, Evolution, Genetics, Environmental social sciences, Ocean sciences

## Abstract

Despite multiple conservation efforts of the Mexican government, the leatherback turtle is at serious risk of extinction. In this study, we investigated the possible presence of a genetic bottleneck that could prevent the recovery of this species and compared these findings with those of the olive ridley turtle, which is in true recovery. Our results confirmed that a demographic change occurred in the past and the presence of two different leatherback turtle lineages that diverged approximately 13.5 million years ago. Local ecological knowledge (LEK) also described the presence of these two lineages and warned that one is at higher risk of extinction than the other. Genetic analysis confirmed 124 mutations between the two lineages, and much lower genetic diversity in one lineage than the other. Our study highlights and substantiates the power of mixing LEK, environmental history, and genetics to better understand conservation challenges of highly threatened species such as the leatherback turtle. Moreover, we report a new lineage of the leatherback turtle which may represent a distinct species. Future studies should focus on morphological, ecological, biogeographical, evolutionary and conservation perspectives for the analysis of the new lineage.

## Introduction

Despite multiple and ongoing efforts of the Mexican and Costa Rican governments in the eastern tropical Pacific, leatherback turtle (*Dermochelys coriacea* Vandelli, 1761) populations have been in sharp decline for almost three decades, and the species is currently on the verge of extinction^1,2^. Over recent decades, key nesting sites in the Mexican Pacific have been identified and heavily protected to prevent poaching of both the eggs and nesting females, and laid eggs are often moved to protected hatcheries^3^.

In addition to the ever-present threats to nesting females and eggs on beaches, mortality due to high rates of bycatch in the ocean, which mainly occurs as a result of entanglement in longlines meant for swordfish^4^, has been hypothesized to be the most important obstacle preventing the species from recovering^2^. One satellite survey of leatherback turtle individuals found that leatherback turtles from Mexico migrate to the highly productive areas of the south-eastern tropical Pacific to feed, which places them in direct contact with various fishing activities^5^. Nonetheless, the sheer size of the area in which they migrate and the small number of turtles from Mexican beaches that have been tracked (7 from Michoacán in 1997^6^, 14 from Oaxaca, and 12 from Michoacán in 2012^5^) call into question whether the barrier to the recovery of Mexican leatherback turtle populations is in fact oceanic fishing activities.

**Figure 1:**
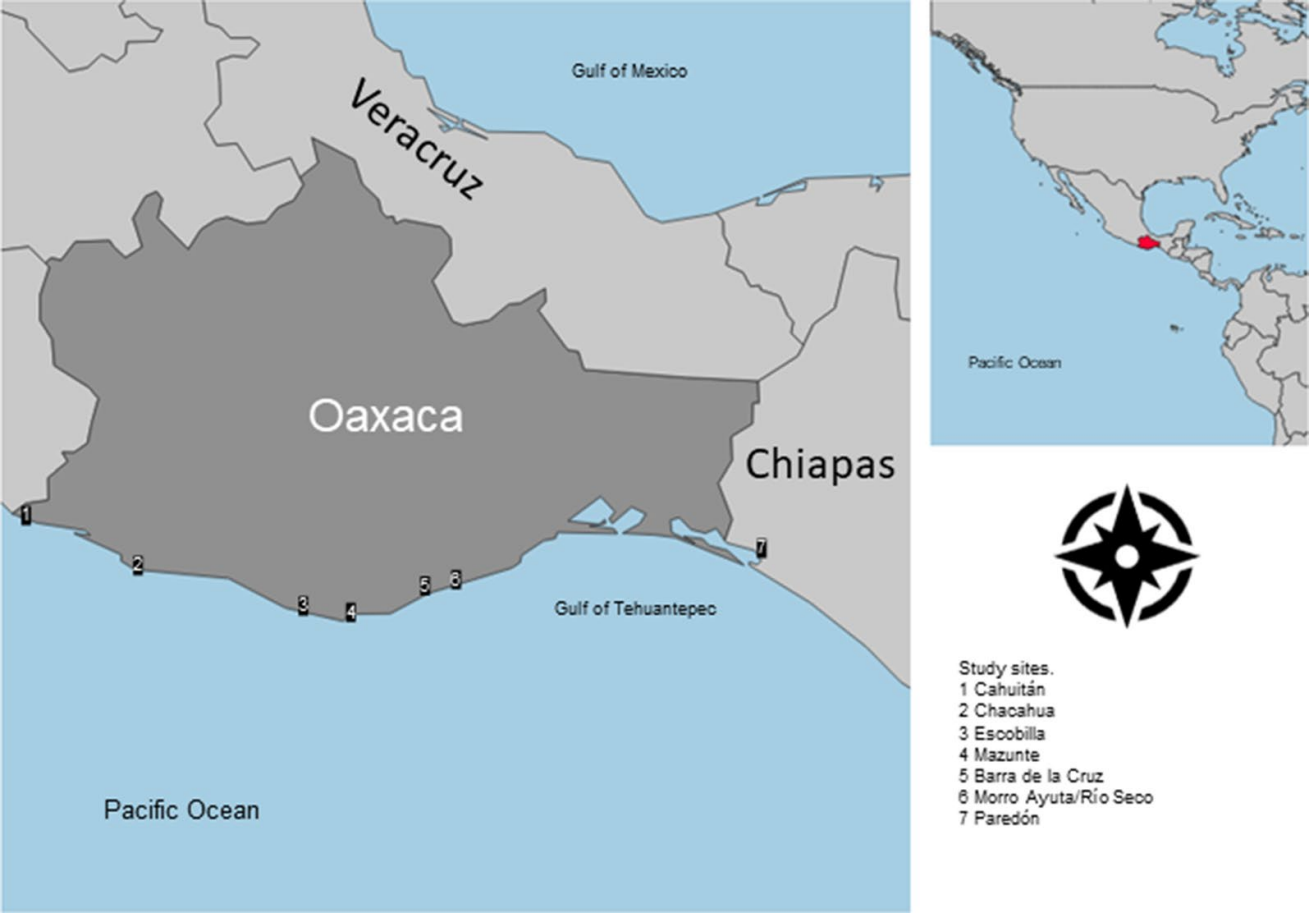
Sampling site and the locations where interviews were conducted. In sites 1 and 5, we sampled tissues from *Dermochelys coriacea*. In sites 3 and 6, we took samples from *Lepidochelys olivacea*. Interviews were conducted in sites 1, 2, 3, 4, 5, and 6.

In contrast to the population trajectory of the leatherback turtle, the population of olive ridley turtles (*Lepidochelys olivacea*, Eschscholtz 1829) from the beaches of Oaxaca (Fig. 1) is clearly recovering^3,7,8^. Like the leatherback turtle, the olive ridley turtle also suffers from the poaching of both eggs and adult females as well as bycatch^8^. For example, the olive ridley turtle is captured in the longline fisheries of Costa Rica with a high annual rate of incidence, which resulted in > 600,000 turtles being caught from 1999 to 2010^9^. Although conservation efforts for both species in the eastern tropical Pacific are similar, their population sizes are different. This led us to ask whether an evolutionary bottleneck could explain the contrasting population trajectories of these species in Oaxaca.

During the nineteenth century, two different species of leatherback turtles were considered to be distributed in the eastern Pacific Ocean^10–12^. However, this notion was discarded due to a lack of evidence^13^. Nowadays, the Dermochelidae family is considered to be represented by only one globally distributed species. For the last three decades, local ecological knowledge (LEK) has been proven to be a powerful tool to speed our understanding of species, ecological life histories, and population trajectories, especially in the marine realm^14–17^. In addition, population genetics has proven to be a powerful tool to uncover some biological and ecological information, particularly for rare and endangered species^18–20^. Although combining genetic information with local ecological knowledge has been an approach rarely used in marine science^21^, it appears to have great potential to better understand barriers that are limiting the recovery of marine animals threatened for their conservation in moderns times. Here we propose to undertake this combined approach to better understand the demographic trajectories for the leatherback and olive ridley turtles.

In our study, we collected local ecological knowledge to identify threats that could be preventing the recovery of the leatherback turtle that could have passed unnoticed in western literature. To our surprise, the first barrier identified by local people regarding the conservation of this species was that modern ecological monitoring did not recognize what was pretty obvious to local people—that there were two different types of leatherback turtle (Table 1) and that one of them was more endangered than the other. By analyzing samples to identify evolutionary bottlenecks, we were able to corroborate the presence of two lineages for the leatherback turtle, which we report in this paper.

**Table 1:** Genetic diversity of nesting females of *Lepidochelys olivacea* and *Dermochelys coriacea* on the beaches of Oaxaca, Mexico (Fig. 1), and for two lineages identified for *D. coriacea*.

Species/lineage	Beach	*N*	*h*	*Hd*	Nucleotide diversity ± SD	Polymorphic sites	*θ*_*W*_	*Fs*	*D*
*Leatherback*	Barra de la Cruz	14	4	0.582	0.07480 ± 0.01895	124	0.05349	21.514	1.68452 P > 0.10
	Cahuitán	10	3	0.622	0.09121 ± 0.01620	125	0.06061	20.376	2.50786 P < 0.01
	Total	24	5	0.59	0.07917 ± 0.01186	125	0.04592	31.92	2.77964 P < 0.01
*Leatherback lineages*	Lineage A	16	2	0.125	0.00034 ± 0.00029	2	0.00082	0.177	− 1.49796 P > 0.1
	Lineage B	8	3	0.714	0.00176 ± 0.00046	3	0.00158	0.671	0.45766 P > 0.10
*Olive ridley*	Escobilla	12	4	0.636	0.00119 ± 0.00032	3	0.00159	− 1.256	− 0.82879 P > 0.1
	Morro Ayuta	13	4	0.526	0.00094 ± 0.00032	3	0.00155	− 1.658	− 1.233 P > 0.1
	Total	27	5	0.584	0.00109 ± 0.00024	4	0.00166	− 1.75	− 0.90164 P > 0.1

*N* sample size, *h* number of haplotypes, *Hd* haplotype diversity, *SD* standard deviation, *θ*_*W*_ Watterson’s theta, *Fs* Fu’s *Fs* statistic, and *D* Tajima’s D statistic.

## Results

### Genetic analyses

#### Genetic diversity

Values of genetic diversity obtained for the leatherback turtle were notably higher than those that have been previously reported^22–24^. A total of 124 and 125 polymorphic sites were found in Barra de la Cruz and Cahuitán, respectively. A total of five haplotypes were found for both localities (three in Cahuitán and four in Barra de la Cruz). The nucleotide diversity was higher in Cahuitán (*π* = 0.091) than in Barra de la Cruz (*π* = 0.0748), although the same tendency was observed with haplotype diversity (*Hd*) and Watterson’s theta (*θ*_*W*_; Table 1).

When the leatherback results were split into two lineages, the genetic diversity parameters decreased considerably (up to an order of magnitude) and were similar to those of the olive ridley turtle (Table 1). Lineage A (composed of Haplotypes H1 and H5, N = 16) exhibited the lowest level of genetic diversity based on *Hd* and *θ*_*W*_, while lineage B (H2, H3, and H4, N = 8) showed diversity levels similar to those of the olive ridley turtle (Table 1).

In the olive ridley sequences, three and four polymorphic sites were found for each locality and for all samples, respectively. For all samples, nucleotide diversity (*π*) was 0.00109 while *θ*_*W*_ was 0.00166. A total of six haplotypes were found with an *Hd* value of 0.584. We found a higher level of diversity in olive ridley turtles than in lineage A of leatherback turtles but lower than in lineage B based on the *Hd* and *π* data (Table 1).

Fu’s *Fs* values for leatherback lineages A and B were 0.177 and 0.671, respectively, whereas the *Fs* value for the olive ridley turtle was − 1.75. In addition, Tajima’s *D* was not significant for lineage A (− 1.497), lineage B (0.457), or the olive ridley turtle (− 0.901).

#### Haplotype network

**Figure 2:**
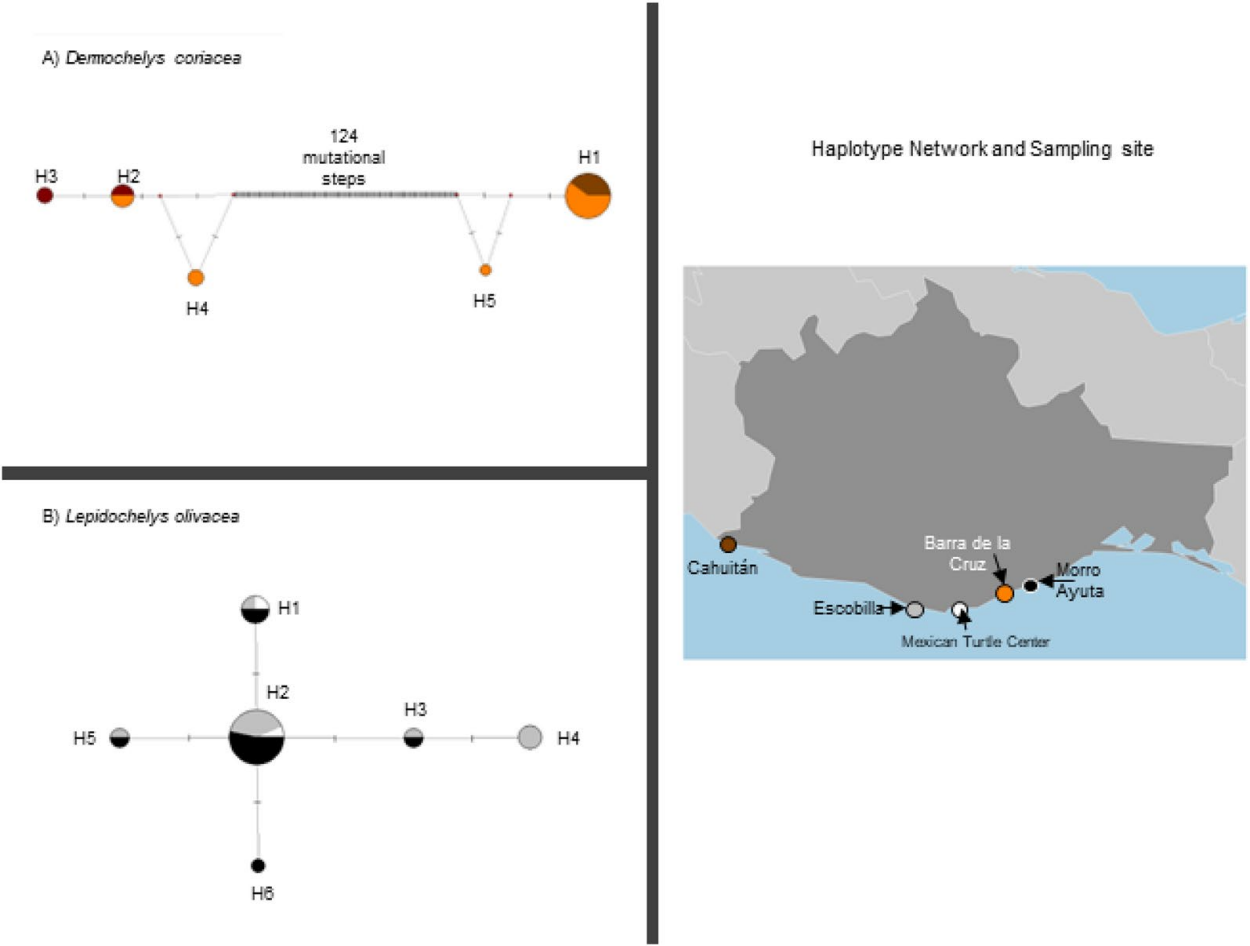
Haplotype networks for (**A**) *Dermochelys coriacea* and (**B**) *Lepidochelys olivacea*. Each color corresponds to a sampling site depicted in the right panel. The size of each circle is proportional to the number of individuals per population. Mutations are depicted as small transversal lines, and the number at the center of the haplotype network in (**A**) (124) represents the number of mutations between the two lineages.

The haplotype network for the leatherback turtle showed two lineages separated by 124 mutational steps. One lineage was made up of H1 and H5 while the other was made up by H2, H3, and H4 (Fig. 2A). We did not find geographic structure as both lineages were found in each sampled locality. The number of mutations between haplotypes within each lineage was one mutational step.

For the olive ridley turtle, the haplotype network showed a starlike shape with haplotype H2 placed at the center of the network. H2 had the highest frequency (N = 15); this haplotype was found in both beaches and in the Mexican Turtle Center. The second-most frequent haplotype was H1 (N = 4), which was also found in these three locations. Haplotype H4 (N = 3) and haplotypes H3 (N = 2) and H5 (N = 2) were only found in Escobilla. Haplotype H6 (N = 1) was private to Morro Ayuta (Fig. 2B).

#### Molecular clock

**Figure 3:**
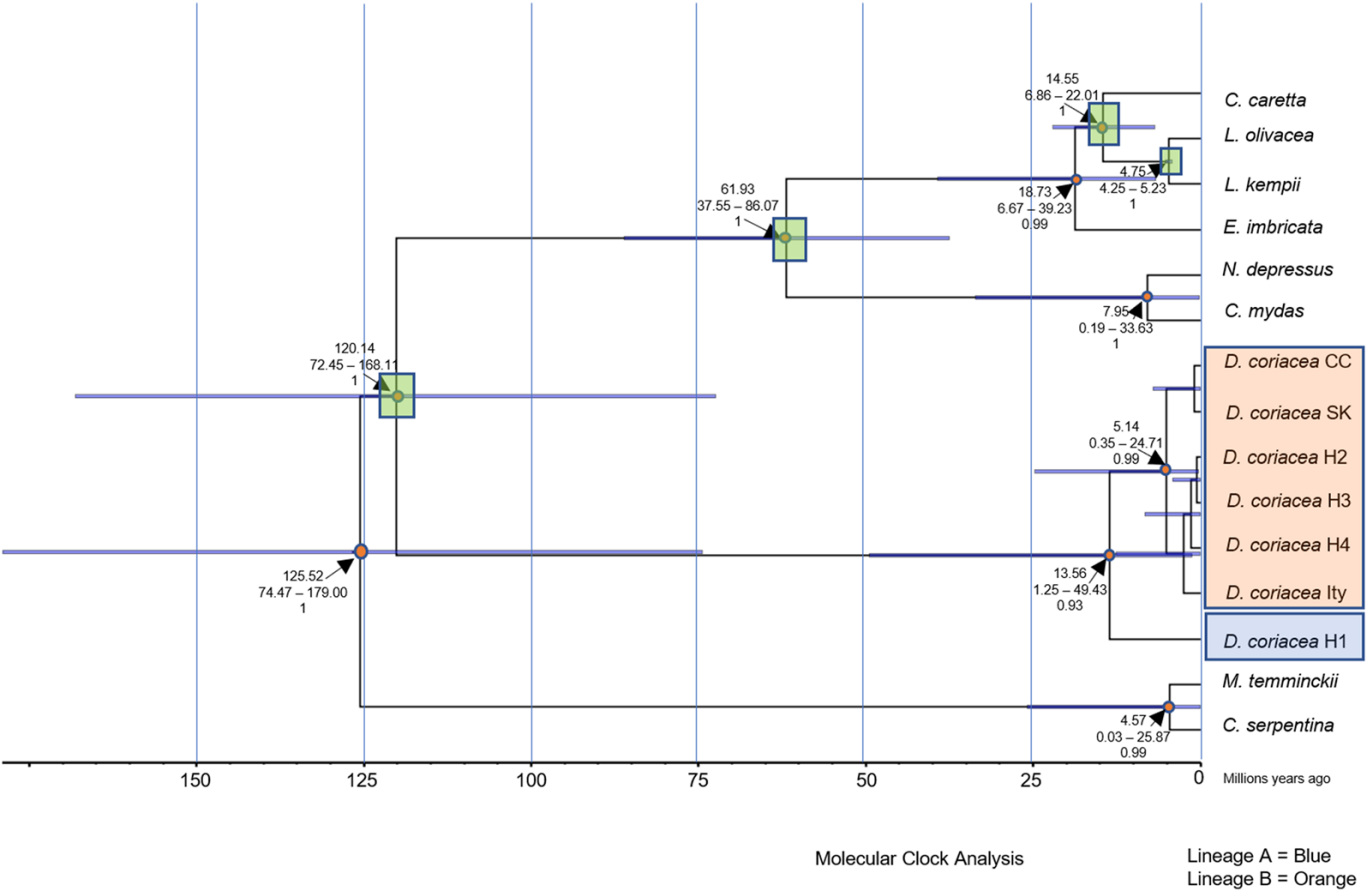
Maximum clade credibility tree showing the divergence times between current sea turtle clades. For each node, the median age, the 95% highest posterior density, and support values > 0.9 are shown. Letter identifiers following *Dermochelys coriacea*: CC = Colombian Caribbean, SK = South Korea, Ity = Italy, H1…H4 (Haplotypes found in our study). Lineage A is highlighted in blue and lineage B is highlighted in orange. The calibration points used in this study are highlighted with green squares.

The independent runs made with Beast2 exhibited posterior values of − 2756.42 and − 2756.09 with ESS values of each parameter > 350. This suggests that the runs converged and that the results are robust. Also, the Maximum Clade Credibility tree (MCC; Fig. 3) obtained in these analyses showed values with high branch support. In the final tree, we obtained a divergence times of 120.14 Mya (72.45–168.11, 95% highest posterior density [HDP], 1 posterior) between the Dermochelyidae and Cheloniidae families; 61.93 Mya (37.55–86.07, 95% HDP, 1 posterior) between species of the Cheloniidae family; 14.55 Mya (6.86–22.01, 95% HDP, 1 posterior) between *Caretta* and *Lepidochelys*; 13.5 Mya (1.25–49.43, 95% HDP, 0.93 posterior) between lineages A and B of *Dermochelys coriacea* found in the present study; 7.95 Mya (0.19–33.63, 95% HDP, 1 posterior) between *N. depressus* and *C. mydas*; and 4.75 Mya (4.25–5.23, 95% HDP, 1 posterior) between *L. kempii* and *L. olivacea.*

#### Community interviews

Of the nine interviewees, four people (two men from Barra de la Cruz and two men from Cahuitán) reported and described having seen a turtle that was distinct but similar to the leatherback turtle (Table 2). The interviewees agreed that a spotless variant exists that is both darker and smaller than the conventional form recognized by the conservation programs and the academic community. One community member mentioned that the hind flippers of the spotless variant were proportionally smaller than those of the conventional form. Another interviewee remarked that the eggs of the spotless variant are smaller and have harder shells than those of the conventional form. All interviewees agreed that the spotless variant is also much less abundant than the conventional form. During our fieldwork, we observed one turtle with characteristics that matched the descriptions of the interviewees of the spotless variant, namely the spotless shell and relatively small hind flippers. Unfortunately, we were not able at the time to take a photograph of this individual. This turtle was registered and found to belong to lineage A.

**Table 2:** Descriptions from expert community members of the turtle variety that is distinct from the leatherback turtle (*Dermochelys coriacea*).

Interviewee	Community	Description
Male, 64 years old	Barra de la Cruz, Oaxaca	"Turtles have come here, several that are all black and several with white spots…the black ones have a *bodoque* (bulging shell) on the top of the shell …"
Male, 78 years old	Barra de la Cruz, Oaxaca	"There is a smaller leatherback turtle that lays less eggs than the other leatherback… The *parlama*^a^ is similar to the leatherback but is black and lays smaller eggs than the leatherback. We used to see 1 or 2 *parlamas* a week, right now I think it doesn’t even come out… [The *parlama*] is the same as the leatherback, but it is smaller and the eggs are also smaller and the shell of the eggs are hard as if they were [made of] plastic; it has a very rough shell, solid as plastic, but it is smaller than the leatherback. The *parlama* is no longer seen; I don’t know if it disappeared or went elsewhere but there is almost none…”
Male, 58 years old	Cahuitán, Oaxaca	"There’s one that’s blacker and doesn’t have all those *huilos* (keels of the shell) …it looks bulkier… there is one that is somewhat squashed and grayish. There are some that are very large."
Male, 35 years old	Cahuitán, Oaxaca	"Yes, there are some (leatherback turtles) that have white spots… because there are some of those that come out all black. Supposedly we know them here because they are surlier than the others. The spotted ones are surlier. The black ones are harder to see and rarely come out here. I think there are very few of those ones because they hardly ever came out.”
Male, 70 years old	Paredón, Chiapas	Here is their sanctuary where is possible to see the *parlama* tigre^b^… There are two types of *parlamas*, one that has spots, and the other spotless but bigger. Both *parlmas* have the turtle shell like skin, very oily; it does not really look like a hard shell. *Parlama* toro is wider, much bigger…
Male, 67 years old	Paredón, Chiapas	We haven't seen the *parlama* toro in years, well sometimes it can be seen. The *parlama* tigre is thinner. The *parlama* toro is bigger it has a taller back

^a^*Parlama* is sometimes used as the common name for leatherback turtle in Oaxaca and Chiapas states, especially by elder people. More recent generations used parlama as generic for all marine turtles.

^b^In Chiapas tiger (tigre in Spanish) is the common name for jaguar (*Panthera onca*). Toro means bull (*Bos taurus*) in Spanish.

## Discussion

In the present study, we used a LEK and population genetics approach to better understand the demographic trajectories of the leatherback and the olive ridley turtles. Our combined approach allowed uncovering an undescribed lineage for the leatherback turtle, which may be in high risk of extinction. Thus, our study highlights the importance of considering local knowledge in the study of biodiversity and corroborating this information with appropriate methods.

We registered lower levels of genetic diversity in terms of polymorphic sites, *π*, and *Hd* for *L. olivacea* compared to those of other studies^25^. In contrast, we found high genetic diversity levels for *D. coriacea*. This was not expected given that the population of *D. coriacea* is currently decreasing while that of *L. olivacea* is increasing^3,8^. The values of nucleotide diversity for the leatherback turtle (*π* = 0.07917) were 72 times higher than the value for *L. olivacea* (*π* = 0.00109) and higher than those reported by Dutton et al.^22^ (*π* = 0.0015), Vargas et al.^24^ (*π* = 0.0011), and Dutton et al.^23^ (*π* = 0.0006–0.0032). These high levels of genetic diversity were clearly caused by nucleotide differentiation between the two groups of haplotypes (124 polymorphic sites). When we considered each lineage separately, the nucleotide diversity values were consistent with those obtained for the olive ridley turtle and with those that have been previously reported for the leatherback^22–24^.

The haplotype diversity value for lineage B (*π* = 0.714) is very similar to the value reported by Dutton et al.^22^ (*π* = 0.712) for Mexiquillo beach in the Mexican Pacific. The nucleotide diversity of Lineage A (0.00034) was an order of magnitude lower than that of lineage B (0.00176), which concurs with what has been reported previously^22–24^. These values suggest that lineage A may be less diverse than lineage B, and thus individuals from lineage A may be more vulnerable than leatherback turtles from lineage B.

The latter is further supported by interviewees reporting one morphotype that is rarely seeing.

The high and surprising genetic diversity of *D. coriacea* was associated with the presence of two lineages that are genetically distinct and reflect different evolutionary histories and demographic changes. The interviewees recognized the presence of a leatherback turtle variant, which was supported by the genetic information generated in this study. The results suggest the presence of a different species or subspecies of leatherback turtle on the beaches of Oaxaca that diverged ~ 13.5 Mya. Nonetheless, further morphological analyses are necessary to confirm that this lineage constitutes a distinct species, along with genetic analyses based on nuclear molecular markers such as microsatellite loci or single nucleotide polymorphisms to test for reproductive isolation between the two lineages. In addition, one interviewee mentioned differences in the number of eggs laid for each morphotype, as well in the size of the eggs and the hardness of the eggshell. These observations have to be considered in future study designs in order to be confirmed. On the other hand, the values of *Hd* (0.584) and *π* (0.00109) for *L. olivacea* are lower than those that have been previously reported in other locations of the Mexican Pacific (*Hd* = 0.6048 and *π* = 0.0022 in Ceuta, Sinaloa; *Hd* = 0.6190 and *π* = 0.0023 in El Verde, Sinaloa; and *Hd* = 0.6800 and *π* = 0.0029 in Ixtapilla, Michoacán)^25^. The lower levels of genetic diversity found in this study could be related to the exploitation of this species in the region. Even if the populations of this species are currently increasing, the recovery of genetic diversity after a bottleneck would depend on the magnitude and duration of a bottleneck^26^. As marine turtles have long generation times, their effective population size, and their genetic variation, would take a longer time to recover.

Fu’s *Fs* values for leatherback lineage A (0.177) and lineage B (0.671) are consistent with a signal from a population bottleneck. In contrast, the olive ridley turtle value (− 1.75) agrees with those expected for a species experiencing a recent population expansion. and is further supported by a starlike shape in the haplotype network. Tajima’s *D* on the other hand was not significant for either leatherback lineage or for the olive ridley turtle. Nevertheless, Tajima’s *D* is not as sensitive to demographic changes as Fu’s *Fs*, thus a positive value (even if not significant) for both leatherback lineages supports the existence of a historic bottleneck, while the negative value for the olive ridley turtle suggests population expansion^27^.

In the case of the haplotype networks for the leatherback turtle, large genetic distance was observed between lineage A (reported for the first time in this study) and lineage B (previously reported with data collected in different areas of the world; Fig. 2). Both lineages are distributed on Barra de la Cruz and Cahuitán. The lack of resolution in the haplotype network within each of the two leatherback lineages (observed loops) may be related to the high mutation rate for the mtDNA D-loop and the small sample size given that we are describing two different lineages. We recommend increasing the sample size of both variants, including samples from Central America, and to use other molecular markers for better resolution. The haplotype network of the olive ridley turtle showed a star-like shape, which, as previously mentioned, has been related to population expansions.

The dates of divergence from the molecular clock were consistent with those previously reported by Duchene et al.^20^. The molecular clock analysis suggests that leatherback lineages A and B diverged approximately 13.5 Mya (Fig. 3). This event is older than the divergence times of other sister species, such as those in the *Lepidochelys* genera, which occurred ~ 4.75 Mya, and those of *C. mydas* and *N. depressus*, which separated ~ 7.95 Mya. However, this divergence time is similar to that of the *Caretta* and *Lepidochelys* genera (14.55 Mya). The molecular clock analysis also revealed that the haplotypes of lineage B in the eastern Pacific are closely related to those found in the Italian (Ity), South Korean, and Colombian Caribbean (CC) areas, whereas lineage A has evolved as an independent group (Fig. 3). Nevertheless, since mtDNA is maternally inherited, we cannot rule out the possibility of gene flow or introgression between these groups until nuclear genetic markers are analyzed. The Eastern Tropical Pacific extends from the Gulf of California to Peru, and encompasses four biogeographic regions: the Cortez Province, the Mexican Province, the Panamic Province and the Galapagos Province^28^. Our sampling area falls within the Gulf of Tehuantepec, which represent the southern limit of the Mexican Province. Future studies should delineate the distribution of each lineage and determine if the two lineages are sympatric south of the Gulf of Tehuantepec or they are allopatric, and the coast of Oaxaca constitutes an area of range overlap.

In his “Natural History of the Reptiles of Bermuda,” Garman^10^ mentioned that leatherback turtles from different oceans were morphologically distinct. Garman described two different species of leatherback turtle, *Sphargis coriacea* (*Sphargis* now correspond to *Dermochelys*) and *S. schlegelii*, although no descriptions were given for *S. schlegelii.* Later, one *S. schlegelii* specimen was included in a herpetological list of the Isthmus of Tehuantepec^11^, which is in the same region as our study area.

In 1899, Philippi^12^ proposed a new species of *Sphargis* nominated *S. angusta*. The specimen was collected near Tocopilla, Chile. The description of this new species indicated that the shell was narrower and darker than that of *S. coriacea* with spots that were yellowish or hardly visible. The description also indicates that the hind flippers were proportionally smaller than those of *S. coriacea* while the neck and tail were longer and the back flippers pointier. The reptile collection of the National Museum of Natural History of Chile has embalmed specimens of *D. angusta* and *D. coriacea* that were preserved in 1916^29^. Until the first half of the twentieth century, *D. schlegelii* was described as a valid species^30,31^.

Pritchard^13^ compiled taxonomic descriptions of both species and considered that there was not enough information to continue referring to them as separate species and thus advised that they should collectively be classified as *D. coriacea*. More recently, Eckert et al.^32^ noticed the taxonomic ambiguities that have arisen since 1884 and concluded that no author had made a sufficiently valid claim to either confirm or reject the existence of two different species or subspecies of leatherback turtle. Some authors argue that the morphological differences may have been the result of adaptations to environmental conditions or simple variations between populations^22,32^. Future research should search for preserved specimens in herpetological scientific collections, so that morphological and genetic data can be correlated, and to confirm the taxonomic status of this taxon.

The results of the genetic diversity analysis, haplotype network, molecular clock analysis, and interviews suggest the existence of two variants of leatherback turtles. However, it is still too early to define if they are two separate species. The morphological characteristics described by local people of both variants agree with older accounts that describe what were considered to be two different species of leatherback turtle in the past (Table 1)^10–12^. Thus, it is urgent to conduct a taxonomic reassessment of the leatherback turtle that includes both population genetics analyses and morphological descriptions of each specimen. Sufficiently robust sampling efforts are needed that cover large areas to determine whether we are currently dealing with one highly endangered leatherback turtle species or two different species. Nuclear genetic analyses are also needed to understand whether both groups have remained separate over 13.5 million years or there has been secondary contact or introgression.

Regarding current population trends and population recovery of these turtles, the threat that the Chilean swordfish fishery^5^ potentially poses to the entire eastern tropical Pacific leatherback turtle population appears to be only one component of a complex network of factors that currently influence the potential recovery of these sea turtle lineages. The results of our study clearly highlight the need to invest more time and resources to study the ecology, demography, evolutionary history and population genetics of these sea turtles. With better information, we will be able to infer the evolutionary trajectories of both leatherback lineages with more confidence and the potential capacity of the olive ridley turtle to increase its population size even with apparently low levels of genetic diversity.

Conservation genetics/genomics have been largely recognized as a key tool to improve conservation strategies^33–35^ along with local ecological knowledge^15,17,36^. However, combining local ecological knowledge with genetics appears to be an untapped tool to better understand the dynamics that are impeding the recovery of highly endangered marine animals such as marine turtles. However, global studies have also found similar agreement. For example, Dao et al.^21^ identified that local people understand the potential genetic connectivity of marine species through ocean currents.

Our study shows, how genetic analyses coupled with LEK, can help us speed our understanding of the complex drivers that are behind the recovery of vulnerable species such as the leatherback turtle in the eastern Pacific. We are aware there is still work to be done to determine if there are indeed two separate species of leatherback turtle. Nonetheless, the information collected in our study indicates that the conservation strategies aimed at the recovery of this species in the Eastern Pacific Ocean will benefit from understanding its evolutionary trajectory and the observations made by local people.

## Methods

### Study site

The state of Oaxaca is located in southern Mexico (Fig. 1). The coastline of Oaxaca extends into the Gulf of Tehuantepec, which is characterized by strong upwelling and highly productive waters during winter. The gulf is also one of two biological action centers in the eastern tropical Pacific^37^. For this study, we collected tissue samples from turtles in four beaches of Oaxaca: Cahuitán, Barra de la Cruz, Escobilla, and Morro Ayuta (Fig. 1). These beaches are considered some of the most important nesting beaches for leatherback and olive ridley turtles in the state of Oaxaca.

In 2021, we collected skin tissue samples from olive ridley turtles in July in Escobilla and Morro Ayuta. The leatherback turtle samples were collected in November and December in Cahuitán and Barra de la Cruz. We collected 15 tissue samples from each site for each species. To standardize the methodology used to collect tissues and to extract and amplify DNA, we collected two additional tissue samples from olive ridley turtles kept in the Mexican Turtle Center in Mazunte, Oaxaca.

All samples were collected following the rules and procedures established in the Mexican Norm (NOM-162-SEMARNAT-2012) to manage marine turtles in their nesting sites, which includes all ethical and conservation standards for handling sea turtle species considered to be at risk in Mexican waters. A sampling permit was expedited by *Direccion de Vida Silvestre* from the *Secretaria de Medio Ambiente y Recursos Naturales* (permit number SGPA/DGVS/02140/21). All ecological methods were performed in accordance with the relevant guidelines and regulations.

The skin of each turtle was cleaned with an antibenzil solution before and after taking each sample. We used a 6-mm diameter biopsy punch to collect each tissue sample from the hind flipper. Tissue samples were stored in 1.5 ml microcentrifuge tubes with 90% alcohol and transported to the genetics laboratory of *El Colegio de la Frontera Sur* (ECOSUR). Samples were stored at − 20 °C until DNA extraction. We used existing registration numbers to identify each female before sampling and to ensure that no female was sampled twice.

### DNA extraction, amplification, and sequencing

DNA extraction was performed using the phenol:chloroform: isoamyl alcohol method^38^. The mtDNA control region (D-loop) was amplified via PCR with primers H950g (5′-GTCTCGGATTTAGGGGTTTG-3′) and LCM15382 (5′-GCTTAACCCTAAAGCATTGG-3′) for the leatherback turtle and H950 (5′-GTCTCGGATTTAGGGGTTTG-3′) and LTEi9 (5′-AGCGAATAATCAAAAGAGAAGG-3′) for the olive ridley turtle that were designed by Abreu-Grobois et al.^39^. For the PCR, the tubes were prepared with 12 μl of Taq PCR Master Mix (QIAGEN, Hilden, Germany), 10 μl of nuclease free water, 2 μl of each primer at 10 nM, and 2 μl of DNA (~ 20 ng/μl). We added nuclease free water instead of DNA to one of the reactions in each batch as a negative control.

We modified the amplification protocol proposed by Dutton et al.^23^ with an initial denaturation step at 94 °C for 5 min, followed by 35 cycles with denaturation at 94 °C for 30 s, annealing at 53 °C for 1 min, extension at 72 °C for 2 min, a final extension step at 72 °C for 10 min, and hold at 4 °C ad infinitum. For the olive ridley turtle samples, the program consisted of 94 °C for 5 min, followed by 35 cycles with denaturation at 94 °C for 45 s, annealing at 52 °C for 45 s, extension at 72 °C for 45 s, a final extension step at 72 °C for 5 min, and hold at 4 °C^40^. DNA extraction and amplification success were assessed by agarose gel electrophoresis and visualized with ethidium bromide.

In all, 24 leatherback turtle samples (10 from Cahuitán and 14 from Barra de la Cruz), and 27 olive ridley turtle samples (2 from the Mexican Turtle Center, 12 from Escobilla, and 13 from Morro Ayuta) were successfully amplified. Nucleotide sequences were obtained from PCR products, using the forward primer, through the Sanger method in a ABI 3730xl DNA analyzer (Applied Biosystems, Waltham, USA) at Macrogen Korea (dna.macrogen.com).

### Genetic diversity

All sequences were evaluated for quality, and those with poor quality were discarded. Subsequently, a BLAST analysis was conducted to confirm that the correct region was amplified. The sequences were aligned and edited with BioEdit^41^. Sequence lengths were 732 and 625 bp for leatherback and olive ridley turtles, respectively. Basic genetic diversity measures were calculated, namely the number of haplotypes (*h*), haplotype diversity (*Hd*), nucleotide diversity (*π*), and Watterson’s theta (θ_W_). We also evaluated neutrality with Fu’s *Fs* and Tajima’s *D* in DnaSP v. 6^42^ to infer the historical demography for each species; negative values suggest population expansion while positive values suggest a demographic bottleneck^27^.

### Haplotype network and molecular clock

The haplotype network was obtained with Network 10 using the median joining method^43^. To estimate the divergence time and the phylogenetic relationship of the new lineage found in this study, we obtained the substitution model that best fit with our data with jModelTest^44^ based on the Akaike criterion. The model with the best fit was HKY.

We conducted a molecular clock analysis to estimate the divergence time with our sequence data. This analysis was carried out with Beast2^45^. We downloaded three more sequences from GenBank for the leatherback turtle with distinct geographic origins in South Korea (East Sea; accession number MF460363), Colombia (Caribbean Sea; MT050522), and Italy (MK674798; this turtle may have had an Atlantic origin according to the authors^46^). We also included sequences for loggerhead (*Caretta caretta*; MT506634), green (*Chelonia mydas*; OK324138), hawksbill (*Eretmochelys imbricata*; DQ177341), Kemp’s ridley (*L. kempii*; MZ043572), and flatback (*Natator depressus*; MN029107) turtles in this analysis. For the olive ridley turtle, we used the most frequent haplotype obtained from this study. As an external group, we used the terrestrial common snapping turtle (*Chelydra serpentina*; EF122793) and alligator snapping turtle (*Macrochelys temminckii*; NC_009260), as suggested by Duchene et al.^20^. These sequences were also obtained from GenBank.

To define calibration points, we followed the methodology of Bowen et al.^47^ and used four calibration points based on the fossil record, which were previously used to date the phylogeny of sea turtles: Cheloniidae and Dermochelyidae families (100–150 Mya^48,49^), Cheloniidae species (50–75 Mya^48,50^), *Caretta* and *Lepidochelys* (12–20 Mya^49,51^), and *Lepidochelys* (4.5–5 Mya^52,53^). All calibration points were set with normal distributions, and we performed preliminary runs to adjust the parameters of the model. Finally, we conducted two independent runs with 100 million MCMC chains and a 10% burnin. We implemented a relaxed log normal clock model and a calibrated Yule model. The results of both runs were combined with LogCombiner^45^, and we obtained the MCC with median height with TreeAnotator^45^. The MCC was visualized with Figtree v. 1.4.4 (available at http://tree.bio.ed.ac.uk/software/figtree/).

### Semi-structured interviews

We conducted interviews with local people that were actively involved in sea turtle conservation or exploitation or that had been in the past. When our first interviewee mentioned a leatherback turtle with differences in size, color, and egg characteristics, we decided to interview different local experts who would have collected eggs before the activity became illegal. We asked these individuals if they were familiar with the variety of leatherback turtle described by the first interviewee. In all, we conducted nine interviews in the communities of Cahuitán, Chacahua, Mazunte, Barra de la Cruz, and Río Seco /Morro Ayuta beach in Oaxaca and Paredón in Chiapas (Fig. 1). Methods involving human subjects were revised and approved by the *Comité de Ética en la Investigación* of ECOSUR on 24 February 2021. All social methods were performed in accordance with the relevant guidelines and regulations.

All the interviews were conducted in the homes of the interviewees or in landing sites. Prior to each interview, we clearly explained the objective of our research. In addition, we ensured the anonymity of any information collected, and informed consent was obtained from all interviewees before the interview formally started. We included questions to assess changes in leatherback abundance and questions regarding the importance of beaches and the leatherback in the dietary habits of local people. The interviews followed a semi-structured format (Appendix I).

## Supplementary Information

Supplementary Information.

## Data Availability

The mtDNA control region sequence data are deposited in GenBank under embargo until the paper is accepted for publication [accession numbers: OP716909 to OP716919].
