# Supplementary material for: Mitochondrial DNA and local ecological knowledge reveal two lineages of leatherback turtle on the beaches of Oaxaca, Mexico
Source: Sci Rep. 2023 May 31;13:8836. doi: 10.1038/s41598-023-33931-4 (PMC10232504; doi:10.1038/s41598-023-33931-4)
Supplement: Supplementary file 1 — Supplementary Information. [file 41598_2023_33931_MOESM1_ESM.docx]

**Appendix I. Interview Format**

**I. General Data**

1. Date (dd/mm/yyyy):
2. Name of interviewer:
3. Name of the interviewee (For archival purposes only):
4. Age:
5. Birthplace (City and Country):
6. Gender:
7. Where do you live?
8. How many years have you lived in this place?
9. How many generations of your family have lived here?

**II. Turtles**

1. Have you ever caught a **Leatherback Sea Turtle** (*Dermochelys coriacea*)?
   1. If your answer was yes:
   2. Can you tell us how you used the leatherback turtle meat?
   3. How was it cooked?
   4. Was the meat sold? Where was it sold?
   5. y what name was leatherback turtle meat known?
   6. What names do you use for the leatherback sea turtle in the region?
   7. Do you know if there is only one leatherback turtle or are there a few variations?

If your answer is YES

- 1. Can you describe the other variation?
  2. What size was the largest leatherback you have caught?
  3. How many leatherback turtle have you catch on your best day? _____cm ____kg
  4. What year was it?
  5. Where was it?
  6. How many turtles did you catch on your best day?
  7. What month was it?
  8. In what year?
  9. Where was it?

1. Have you ever collected leatherback eggs?
   1. How were leatherback turtle eggs called?
   2. Where were they sold? Where were they sold?
   3. How much were they sold for?
   4. How were it cooked?
   5. On their best harvest night, how many eggs did you collect?
   6. How were the eggs preserved?
2. Which was the most important beach for the nesting of the Leatherback turtle?
   1. How many leatherback turtles did you see in one night on that beach?
   2. What year was it?
